# New Insights on Iron Study in Myelodysplasia

**DOI:** 10.4274/tjh.2012.0154

**Published:** 2014-12-05

**Authors:** Noha M. El Husseiny, Dina Ahmed Mehaney, Mohamed Abd El Kader Morad

**Affiliations:** 1 Cairo University Faculty of Medicine, Department of Clinical Hematology, Cairo, Egypt; 2 Cairo University Faculty of Medicine, Department of Chemical Pathology, Cairo, Egypt

**Keywords:** Hepcidin, Myelodysplasia, iron overload

## Abstract

**Objective:** Hepcidin plays a pivotal role in iron homeostasis. It is predominantly produced by hepatocytes and inhibits iron release from macrophages and iron uptake by intestinal epithelial cells. Competitive ELISA is the current method of choice for the quantification of serum hepcidin because of its lower detection limit, low costs, and high throughput. This study aims to discuss the role of hepcidin in the pathogenesis of iron overload in recently diagnosed myelodysplasia (MDS) cases.

**Materials and Methods:** The study included 21 recently diagnosed MDS patients and 13 healthy controls. Ferritin, hepcidin, and soluble transferrin receptor (sTFR) were measured in all subjects.

**Results:** There were 7 cases of hypocellular MDS, 8 cases of refractory cytopenia with multilineage dysplasia, and 6 cases of refractory anemia with excess blasts. No difference was observed among the 3 MDS subtypes in terms of hepcidin, sTFR, and ferritin levels (p>0.05). Mean hepcidin levels in the MDS and control groups were 55.8±21.5 ng/mL and 19.9±2.6 ng/mL, respectively. Mean sTFR was 45.7±8.8 nmol/L in MDS patients and 31.1±5.6 nmol/L in the controls. Mean ferritin levels were significantly higher in MDS patients than in controls (539.14±83.5 ng/mL vs. 104.6±42.9 ng/mL, p<0.005). There was a statistically significant correlation between hepcidin and sTFR (r=0.45, p=0.039). No difference in hepcidin levels between males and females was observed, although it was lower in males in comparison to females (47.9±27.6 vs. 66.7±35.7, p>0.05).

**Conclusion:** Hepcidin may not be the main cause of iron overload in MDS. Further studies are required to test failure of production or peripheral unresponsiveness to hepcidin in MDS cases.

## Abstract (tr)

**Amaç:** Hepsidin demir dengesinde önemli bir rol oynar. Temel olarak hepatositler tarafından üretilir ve intestinal epitel hücrelerinden demir alımını ve makrofajlardan demir salınımını inhibe eder. Kompetitif ELİZA en düşük düzeyleri saptayabilmesi, düşük maliyeti ve yüksek test kapasitesi ile kantitatif serum hepsidin miktari ölçümü için tercih edilen mevcut bir yöntemdir. Bu çalışmanın amacı son zamanlarda miyelodisplazi (MDS) tanısı almış olgularda demir yükünün patogenezinde hepsidinin rolünü tartışmaktır.

**Gereç ve Yöntemler:** Bu çalışmaya 21 yeni tanı MDS hastası ve 13 sağlıklı kontrol alındı. Ferritin, hepsidin ve soluble transferrin reseptörü (sTFR) tüm olgularda ölçüldü.

**Bulgular:** Hiposellüler MDS’li 7 olgu, multilineage displazili refrakter sitopenili 8 olgu ve artmış blastlı refrakter anemili 6 olgu vardı. Bu 3 MDS alt tipleri arasında hepsidin, sTFR ve ferritin düzeyleri açısından fark gözlenmedi (p>0,05). Ortalama hepsidin seviyesi sırasıyla MDS grubunda 55,8±21,5 ng/mL ve kontrol grubunda ise 19,9±2,6 ng/mL idi. Ortalama sTFR, MDS grubunda 45,7±8,8 nmol/L ve kontrol grubunda ise 31,1±5,6 nmol/L idi. Ortalama ferritin seviyeleri ise MDS grubunda kontrolden anlamlı olarak yüksekti (539,14±83,5 ng/mL vs. 104,6±42,9 ng/mL, p<0,005). Hepsidin ve sTFR arasında istatistiksel anlamlı bir ilişki vardı (r=0,45, p=0,039). Hepsidin düzeyleri erkeklerde kadınlarla karşılaştırıldığında daha düşük değerlerde bulunmasına karşılık erkek ve kadınlar arasında istatistiksel anlamlı fark görülmedi (47,9±27,6 vs. 66,7±35,7, p>0,05).

**Sonuç:** Hepsidin MDS’de aşırı demir yükünün ana nedeni olmayabilir. MDS’li olgularda hepsidine çevresel yanıtsızlık veya üretimi yetersizliğini test edecek başka çalışmalara ihtiyaç vardır.

## INTRODUCTION

Myelodysplastic syndrome (MDS) is a group of clonal hemopoietic disorders characterized by ineffective hematopoiesis, bone marrow dysplasia, and an increased risk of transformation to acute myeloid leukemia. The large majority of patients with MDS are anemic and eventually up to 90% of them require regular transfusions. In 50% to 60% of patients, anemia is severe, with hemoglobin levels below 10 g/dL [1].

The liver polypeptide hepcidin plays a pivotal role in iron homeostasis. In macrophages, it accelerates the degradation of the trans-membrane iron exporter ferroportin mRNA. In intestinal epithelial cells, it is believed to down-regulate divalent metal transporter-1, which is involved in the transfer of iron across the intestinal wall [2].

Previous reports on hepcidin levels in MDS showed conflicting results. Murphy et al. found that urinary excretion of hepcidin was lower in MDS in comparison to the control group and interpreted this finding as evidence for hepcidin’s role in iron overload in MDS [3]. However, others reported contrary findings [4]. In an earlier study, we measured pro-hepcidin in MDS and found it to be significantly lower in comparison to levels in the control group [5]. However, the utility of the prohepcidin assay is controversial [6].

The development of quantification techniques based on mass spectrometry [matrix-assisted laser desorption ionization, surface enhanced laser desorption/ionization time-of-flight mass spectrometry (SELDI-TOF MS), or liquid chromatography tandem mass spectrometry] has shown promising results. However, some of these approaches did not use internal standards for the quantification of hepcidin and are considered semi-quantitative; moreover they require specialized equipment that is not widely accessible [7]. Regarding MDS, there are only a few conflicting data about urinary hepcidin measured by using first generation semi-quantitative techniques [8]. The competitive enzyme-linked immunosorbent assay (ELISA) is currently the method of choice for the quantification of serum hepcidin because of its lower detection limit, low costs, and high throughput [6].

Although prolonged red blood cell transfusion therapy appears to be the main contributor to iron overload in MDS, many patients appear to develop iron overload at an early stage of the disease, before the onset of transfusions. It has been postulated that an altered production of hepcidin, the recently discovered key hormone regulating iron homeostasis, may play a role in this regard [8].

The aim of this study was to find out whether hepcidin plays a key role in the pathogenesis of iron overload seen in recently diagnosed MDS, or whether hepcidin levels are just a consequence of the interaction between ineffective erythropoiesis and blood transfusion.

## MATERIALS AND METHODS

This study included 21 MDS patients recently diagnosed in the Clinical Hematology Unit of Cairo University. All patients who had an MDS diagnosis of less than 6 months entered the study if they were not on iron chelation, had no evidence of infection and no renal or liver impairment, and did not receive more than 10 units of blood. Ethical committee approval of the study was obtained. Each individual in the study signed a consent form. 

Five milliliters of venous blood was obtained from each individual in the study after at least 8 h of fasting. For MDS patients, samples were taken at least 5 days after the last transfusion to avoid acute alterations in the hepcidin level. The samples were centrifuged at 3000 rpm for 10 min to separate sera and stored at -80 °C until analyzed.

Serum levels of hepcidin were determined using a commercially available ELISA kit (DRG Instruments GmbH, Marburg, Germany) according to the manufacturer’s protocol. The 5% to 95% range in apparently normal, healthy adults is between 13.3 and 54.4 ng/mL.

Soluble transferrin receptor (sTFR) concentrations were measured in serum, stored at -80 °C, with the use of a commercially available ELISA kit (Quantikine IVD Soluble Transferrin Receptor ELISA; R&D Systems Europe Ltd., Abingdon, UK). The reference interval is 8.7-28.1 nmol/L.

Serum ferritin was quantified using the DRG ferritin kit (ELISA) kit (EIA-1872; DRG International Inc., Mountainside, NJ, USA). The reference interval in healthy males and females is 20-250 ng/mL and 10-120 ng/mL, respectively.

**Statistics Analysis**

SPSS 17 was used for descriptive analysis and comparisons. Spearman’s test was used for correlation analysis and analysis of variance (ANOVA) was used for the comparison of multiple parameters among different groups. Differences and correlations were considered significant when p<0.05. The Kolmogorov-Smirnov test was performed for normal distributions. After suitability analysis, non-parametric or parametric tests were performed. 

## RESULTS

**Figure 1:**
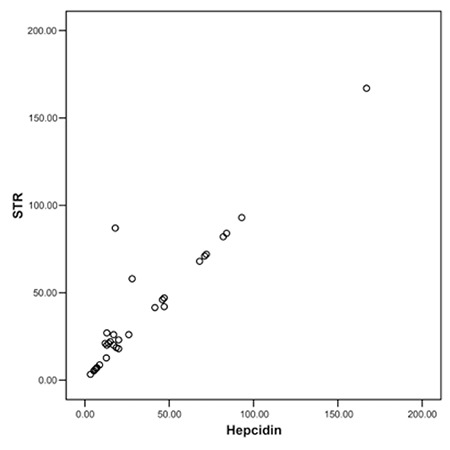
Correlation between hepcidin and sTFR.

This study included 21 MDS patients (10 females, 11 males) recently diagnosed in the Clinical Hematology Unit of Cairo University (average of 6 months after the onset of symptoms) and 13 (6 females, 7 males) age- and sex-matched controls. The mean age was 56±10.2 years. Mean hemoglobin level of MDS patients was 6.8±4.8 g/dL. Median number of blood transfusions was 6 units (range: 3-9 units). Mean hepcidin levels in the MDS and control groups were 55.8±21.5 ng/mL and 19.9±2.6 ng/mL, respectively. Mean sTFR level in MDS patients was 45.7±8.8, while in the control group it was 31.1±0.6. Mean ferritin levels in the MDS patients and the controls were 539.14±83.5 ng/mL and 104.6±42.9 ng/mL, respectively (Table 1). Although the mean hepcidin, sTFR, and ferritin levels were higher in patients with MDS than in controls, only ferritin significantly differed between the 2 groups (p<0.005). Correlation analysis between hepcidin and sTFR was statistically significant (r=0.45, p=0.039, Figure 1). The correlations between hepcidin and other parameters are given in Table 2. There was no statistically significant difference in hepcidin levels between males and females, although levels were lower in males in comparison to females (47.9±27.6 ng/mL vs. 66.7±35.7 ng/mL, p>0.05).

MDS patients were divided according to the type of MDS into 3 groups: refractory cytopenia with multilineage dysplasia (RCMD), 8 patients; hypoplastic MDS, 7 patients; and refractory anemia with excess blasts (RAEB), 6 patients. No statistically significant difference among the 3 groups in terms of hepcidin, sTFR, or ferritin was found (p>0.05, Table 3). Mean hepcidin/ferritin ratio in patients with MDS was higher than in the controls (0.48±1.2 vs. 0.32±0.19), but this was not statistically significant (p=0.6).

## DISCUSSION

It has been reported that ineffective erythropoiesis enhances iron absorption in MDS through down-regulation of hepcidin and its prohormone such that serum ferritin rises to 500-600 ng/mL but seldom exceeds these values before transfusion begins [9,10]. Moreover, MDS is characterized by iron overload secondary to blood transfusion. However, its impact on stimulation of pro-hepcidin release to inhibit iron absorption is less than the erythroid drive suppressing its release, as shown from our earlier results [5].

In this work, we included newly diagnosed (within 6 months of diagnosis) MDS cases to limit the impact of over-transfusion on hepcidin levels. Furthermore, blood samples were drawn at least 5 days after the last transfusion to abolish the effect of acute transfusion on hepcidin expression.

We found hepcidin levels higher in MDS cases in comparison to the control group, but this difference was not statistically significant, which could be attributed to the small number of patients studied.

Our results are in line with those of Qin et al., who found that both hepcidin and serum ferritin levels in MDS patients, regardless of transfusion dependency or the number of blood transfusions, were higher than those of healthy controls [11]. Our findings suggest that iron overload is not related to defective hepcidin release but is rather associated with ineffective erythropoiesis and blood transfusion. 

In another study using SELDI-TOF MS for hepcidin assay, serum hepcidin levels were measured to be slightly higher in MDS patients than in controls, but this difference did not reach statistical significance. Nevertheless, the hepcidin/ferritin ratio was significantly lower for the whole MDS population as compared to the controls, which is not consistent with our study in which the ratio was higher in MDS cases [8]. This could be explained by the higher mean ferritin levels of the MDS population in that study, which also included heavily transfused cases.

Ganz et al. reported that serum hepcidin was high in low-grade MDS patients in correlation with their iron and oxidative status, and that it was further increased by treatment with deferasirox. They concluded that the hepcidin level represented a balance between the stimulating effect of iron overload and the inhibitory effects of erythropoietic activity and oxidative stress. These preliminary findings favor the rationale for iron chelation therapy in such patients [12]. Our results revealed no correlation between hepcidin level and ferritin in patients with MDS in the context of non-heavily transfused patients.

Qin et al. worked on heavily transfused MDS and found that the increase of hepcidin was not in synchrony with the increase in serum ferritin levels secondary to blood transfusion when the number of blood transfusions exceeded 24 U. Hepcidin levels showed a negative relationship to serum ferritin, reflecting the decreased ability of hepcidin to inhibit body iron absorption during the increase of blood transfusion, which finally led to iron overload. Dynamic monitoring of the hepcidin concentration could help in predicting the occurrence of iron overload in transfusion-dependent MDS patients [11].

The current data may indicate that there is no correlation between hepcidin and serum ferritin in MDS cases, and thus hepcidin may not be a main player in iron overload in MDS. A possibility of peripheral unresponsiveness to hepcidin in MDS or failure of production may be the underlying cause, but further studies are required.

When divided into RCMD, RAEB, and hypoplastic MDS groups, the highest levels of hepcidin were in the RAEB group, indicating marked activity in the bone marrow, followed by the RCMD and the hypocellular MDS groups. However, the difference among different subgroups in terms of hepcidin, ferritin, or sTFR level did not reach statistical significance.

Santini et al. found that mean hepcidin levels were consistently heterogeneous across different MDS subtypes, with the lowest levels in refractory anemia with ringed sideroblasts (1.43 nmol/L) and the highest in the RAEB (11.3 nmol/L) (p=0.003) [8].

## CONCLUSION

Iron overload in MDS involves many players. Further studies are required to reveal the causes of failure in erythropoiesis or the peripheral unresponsiveness to hepcidin in MDS cases.

**Acknowledgments**

Special thanks to all members of the Hematology Clinic of Cairo University for their help in collection of data. The researchers were given funding from Cairo University to import ELISA kits for hepcidin assay.

**Conflict of Interest Statement**

The authors of this paper have no conflicts of interest, including specific financial interests, relationships, and/or affiliations relevant to the subject matter or materials included.

## Figures and Tables

**Table 1:**
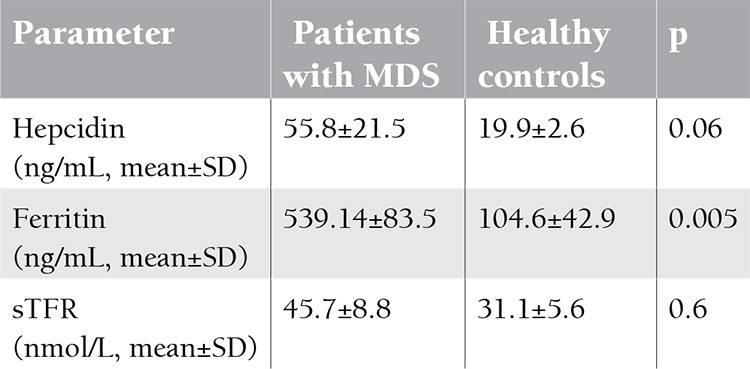
Iron parameters.

**Table 2:**
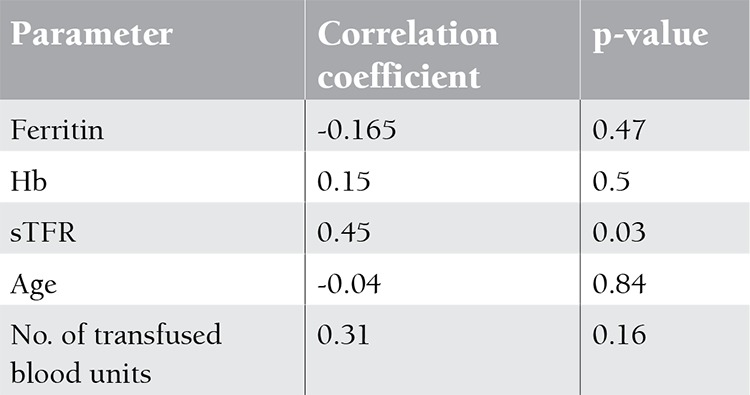
Correlation between hepcidin and other parameters in MDS.

**Table 3:**
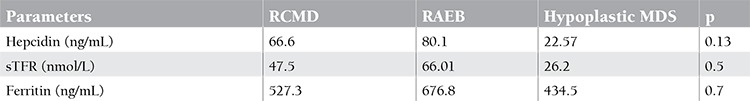
Comparison of different parameters in the 3 main types of MDS.
